# Detecting Medication Risks among People in Need of Care: Performance of Six Instruments

**DOI:** 10.3390/ijerph20032327

**Published:** 2023-01-28

**Authors:** Tobias Dreischulte, Linda Sanftenberg, Philipp Hennigs, Isabel Zöllinger, Rita Schwaiger, Caroline Floto, Maria Sebastiao, Thomas Kühlein, Dagmar Hindenburg, Ildikó Gagyor, Domenika Wildgruber, Anita Hausen, Christian Janke, Michael Hölscher, Daniel Teupser, Jochen Gensichen, on behalf of the BACOM Study Group

**Affiliations:** 1 Institute of General Practice and Family Medicine, University Hospital, LMU Munich, 80336 Munich, Germany; 2 Institute of General Practice, Friedrich-Alexander-University of Erlangen-Nuremberg, 91054 Erlangen, Germany; 3 Institute of General Practice, University Hospital of Julius-Maximilians-Universität Würzburg, 97070 Würzburg, Germany; 4 Katholische Stiftungshochschule München/University of Applied Sciences, 81667 Munich, Germany; 5 Division of Infectious Diseases and Tropical Medicine, Medical Center of the University of LMU Munich, 80802 Munich, Germany; 6 Institute of Laboratory Medicine, University Hospital of LMU Munich, 81377 Munich, Germany

**Keywords:** inappropriate medication, prescribing omission, nursing home residents, polypharmacy, adverse drug reaction

## Abstract

Introduction: Numerous tools exist to detect potentially inappropriate medication (PIM) and potential prescribing omissions (PPO) in older people, but it remains unclear which tools may be most relevant in which setting. Objectives: This cross sectional study compares six validated tools in terms of PIM and PPO detection. Methods: We examined the PIM/PPO prevalence for all tools combined and the sensitivity of each tool. The pairwise agreement between tools was determined using Cohen’s Kappa. Results: We included 226 patients in need of care (median (IQR age 84 (80–89)). The overall PIM prevalence was 91.6 (95% CI, 87.2–94.9)% and the overall PPO prevalence was 63.7 (57.1–69.9%)%. The detected PIM prevalence ranged from 76.5%, for FORTA-C/D, to 6.6% for anticholinergic drugs (German-ACB). The PPO prevalences for START (63.7%) and FORTA-A (62.8%) were similar. The pairwise agreement between tools was poor to moderate. The sensitivity of PIM detection was highest for FORTA-C/D (55.1%), and increased to 79.2% when distinct items from STOPP were added. Conclusion: Using a single screening tool may not have sufficient sensitivity to detect PIMs and PPOs. Further research is required to optimize the composition of PIM and PPO tools in different settings.

## 1. Introduction

Older people are more frequently affected by polypharmacy, and more susceptible to adverse drug reactions (ADR), than younger people due to multimorbidity and physiological aging processes [1,2,3,4,5]. As a guidance for clinicians, a number of consensus-based instruments have been developed listing potentially inappropriate medication (PIM) to be avoided or used with caution in older people. Instruments alerting physicians to potential prescribing omissions (PPO) have also been developed [6,7,8]. Internationally prominent examples include START/STOPP criteria, and EU(7)-PIM, while the PRISCUS and FORTA lists are German developments [9,10,11,12]. More recently, the STOPPFall list has been developed by a European geriatrics society task force, which alerts prescribers to fall risk increasing drugs (FRIDs), while the German-ACB instrument lists drugs with anticholinergic effects available on the German market [13,14].

Application of the PIM and PPO tools listed above could in principle be automated, but in practice this is often limited by a lack of, or incomplete, electronic medical records. On the other hand, the manual application of multiple tools is unpractical, while use of a single tool may miss important PIMs and/or PPOs, raising the question, which tools or combination of tools may provide the best balance of efficiency and comprehensiveness. 

The aim of this study was therefore to compare the above named tools in terms of their overlaps and their coverage of relevant PIMs and PPOs in a convenience sample of older people, alone and in combination. 

## 2. Materials and Methods

### 2.1. Study Design

We conducted a cross-sectional study of older patients in need of care included in the Bayerischer ambulanter Covid-19 Monitor (BaCoM), applying prominent PIM and PPO lists to data collected as part of the study [15]. We examined, (1) the PIM and PPO prevalence according to each instrument, (2) the sensitivity of each PIM/PPO instrument in detecting any PIM and PPO, (3) the proportions of PIMs and PPOs overlapping and exclusively being detected by each instrument, and (4) the additional proportions of PIMs detected when two or more instruments were combined. 

### 2.2. Data Source and Study Population

BaCoM is a multicenter prospective registry study of patients in need of care with three study centers in Bavaria, Germany (LMU Munich, UK Würzburg and FAU Erlangen, registered in the German Clinical Trials Register: DRKS 26039). 

The analyzed BaCoM participants were those with and without a prior history of COVID-19, who had to be at risk of PIM or PPO, and therefore had to be above 65 years of age and take one or more long term medications. They were enrolled by their respective GP or study physicians and in need of care or support. The latter was defined as receipt of financial support by public care insurance according to an officially assessed care level (“Pflegegrad”), or a score of ≥5 on the 7-point Clinical Frailty Scale (CFS) [16,17]. Exclusion criteria were an estimated life expectancy of <6 months (as judged by the recruiting physician), unclear legal residency status, and persons without health insurance. 

Data were collected by trained study assistants, including sociodemographic and health status data to describe the study population. Apart from clinical frailty, the health status also included data on cognitive function (assessed by a Six-Item Screening Tool) and a Montreal Cognitive Assessment Test Blind (MoCA-BLIND) in those with less than three errors in the Six-Item Screening Tool [18,19,20]. Medical diagnoses, medications taken, and vital signs such as blood pressure, heart rate, and forced expiratory volume in 1 s (FEV1) were documented to apply PIM and PPO instruments. Medication schedules and diagnosis lists were either provided by the GP or collected by the study team at the site at which the participant received care, e.g., nursing homes or, in the case of outpatient care, at the participant’s home. The database source therefore partly comprised codes referring to International Statistical Classification of Diseases and Related Health Problems (ICD-coded) diagnosis lists and standardized medication schedules, but also handwritten lists extracted from nursing records. 

### 2.3. Definition of PIMs and PPOs

We included a total of six different instruments designed to detect PIMs or PPOs or both. A brief description of each tool, highlighting the structure, number of items, and data categories required for their application, is provided in Table 1.

All PIM instruments included in this study were applicable to patients aged 65 years or older (without restrictions), and comprised the FORTA list, STOPP, EU(7)-PIM, PRISCUS, German-ACB, and STOPPFall [9,10,11,12,13,14]. From the FORTA list, we only considered medications listed as “C = questionable” and “D = avoid”, according to the authors’ recommendations [12]. 

FORTA, STOPP, EU(7)-PIM, and PRISCUS are generic tools, in the sense that they were designed to cover medication risks across all drug groups, whereas German-ACB [14] and STOPPFall [13] were specifically developed to identify anticholinergic and fall risk increasing drugs (FRIDs), respectively.

In the German-ACB, we only classified as PIM medications with an ACB score of ≥3 [14]. For STOPP-Fall, we considered all 14 drug groups classified as FRIDs, but only defined them as PIM when participants’ risk of falls was increased by one or more of the conditions listed in the accompanying STOPPFall deprescribing tool (e.g., diuretics in the case of hypotension). As PPO tools we included START [9] and FORTA-A (i.e., medications listed as “A = indispensable”). 

### 2.4. Measurement of PIMs and PPOs

All medications were coded using the Anatomical Therapeutic Chemical (ATC) classification and the diagnoses were coded using ICD-10 [21,22]. Where medication doses were required to apply the included PIM/PPO instruments, daily doses were calculated from the instructions provided. When dosage information was missing, these medications were not included in criteria that considered dose. In cases where dosing instructions were “as required”, these were not taken into account in criteria considering only long-term medication. 

Criteria that explicitly considered the duration of intake (e.g., longer than six weeks) were not considered in any patients because this information was not commonly available. Where medical diagnoses were required to apply the respective PIM or PPO instruments, we only considered explicitly documented diagnoses (i.e., did not assume diagnoses based on medication profiles). 

The PIM-defining criteria from each tool were transcribed into a programming language and applied to the data using RStudio V.2022.07.2.

### 2.5. Data Analysis

In order to examine the prevalence of each PIM and PPO instrument, all instruments were first applied separately, and the prevalence was calculated as the proportion of patients (and 95% confidence interval) with one or more respective PIM or PPO. As a result, each medication taken by each patient was classified as a PIM (or not) or a PPO (or not) according to each tool. In order to examine the sensitivity of each PIM and PPO instrument, we defined PIMs and PPOs identified by any of the respective instruments as the gold standard. The sensitivity for each tool was then calculated as the proportion (and 95% confidence interval) of all PIMs/PPOs detected by each respective instrument. Similarly, we calculated the proportion of PIMs/PPOs uniquely detected by each respective instrument, i.e., not by any of the others. The concordance among the different tools was determined by an analysis of interrater reliability using Cohen’s Kappa and overlaps between tools visualized using Venn diagrams [23]. In order to determine which proportions of PIMs/PPOs would be detected by which combination of PIM/PPO tools, we started with the instruments with the highest PIM/PPO prevalence. We then considered, which other tool would detect the most additional PIMs/PPOs not detected by the first tool, etc. The findings were visualized using a Pareto chart. All confidence intervals were calculated using the exact binomial test [24]. 

## 3. Results

### 3.1. Characteristics of the Study Population

Table 2 shows the characteristics of the study population, comprising 226 participants with a median (IQR) age of 84 (80 to 89) years, with most (76.6%) aged ≥ 80 years and about one fifth (22.6%) being ≥ 90 years old. The majority (71.2%) of participants were female, and three quarters (74.6%) were residents of long-term care facilities. 

The median (IQR) score on the CFS was 6 (5 to 7), consistent with moderate frailty, and over half (53.3%) of participants achieved less than 18 points on the MoCA Blind Assessment, consistent with mild cognitive impairment [17,18,19]. The median (IQR) on the Charlson Comorbidity Index (CCI) was 3 (1 to 5), corresponding to moderate severity of comorbidities, and a quarter (26.1%) suffered from severe comorbidities (CCI score ≥ 5) [25]. Three quarters (75.2%) of patients had a documented diagnosis of hypertension, almost a third (31.9%) had atrial fibrillation, and more than 20% were affected by diabetes (27.0%), dyslipidemia (27.9%), and heart failure (23.5%). In addition, 21.2% had a documented diagnosis of depression. 

The median (IQR) number of medications taken concomitantly was 9 (7 to 12). The vast majority of participants (92.5%) received polypharmacy (≥5 drugs), and almost half (45.6%) received excessive polypharmacy (≥10 drugs) [26]. 

### 3.2. Application of PIM and PPO Instruments to Available Data

Of the total 114 criteria of the START/STOPP tool, 91 items (30 items for START and 61 items for STOPP) could be applied. For the remaining 23 criteria, the data required were not available for the sample [27]. Missing but required data includes laboratory data, metrics from medical exams, vital signs from the past, and the date when a diagnosis was made or a medication was prescribed. For FORTA, we excluded vaccination- and cancer-related sections because vaccinations and ongoing chemotherapy were not consistently documented in the available data. For all other instruments, data was available to apply all items. 

#### 3.2.1. Prevalence of PIMs and PPOs

Table 3 shows that considering all PIM tools together, the PIM prevalence (proportion (95% CI) of patients with ≥ 1 PIM) was 91.6 (87.2–94.9)%, 79.6% had two or more PIMs, and more than half (57.1%) had four or more PIMs. However, the PIM prevalence varied considerably by tool, and was highest for FORTA C/D (76.5 (70.5–81.9)%), followed by STOPP (65.9 (59.4–72.1)%), EU(7)-PIM (61.9 (55.3–68.3)%), STOPPFall (36.3 (30.0–42.9)%), PRISCUS (12.8 (8.8–17.9)%) and German-ACB ≥3 (6.6 (3.8–10.7)%). Table 4 shows that the PPO prevalence (percentage (95% CI) of patients with ≥1 PPO) was similar for both instruments used, namely 63.7 (57.1–69.9)% for START and 62.8 (56.2–69.1)% for FORTA A. The prevalence of patients affected by both PIMs and PPOs was 76.1 (70.0–81.5)%.

#### 3.2.2. Sensitivity of PIM and PPO Instruments

PIMs. Table 5 shows that the 226 study participants used a total of 2209 drugs, of which 648 (29.3 (27.4–31.3)%) were identified as PIM by at least one instrument. The most commonly implicated PIMs were psychoanaleptics (13.1%), followed by “other psycholeptics” (12.7%), direct oral anticoagulants (8.0%), and loop diuretics (7.6%). The sensitivity (i.e., the proportion of all PIMs detected by each instrument) of PIM detection was highest for FORTA-C/D (55.1 (51.2–59.0)%), and STOPP (42.4 (28.6–46.3)%), followed by EU(7)-PIM (34.9 (27.3–34.0)%), STOPPFall (19.1 (16.1–22.4)%), PRISCUS (5.6 (3.9–7.6)%), and German-ACB (2.5 (1.4–4.0)%). 

Table 5 also shows a breakdown of PIM detection by the 10 most commonly im-plicated medications (which together accounted for 67.6% of all PIMs identified). FORTA -C/D detected all benzodiazepine and spironolactone PIMs (100.0%) and the vast majority of psychoanaleptic (96.5%), other psycholeptic (80.5%), and betablocker (74.5%) PIMs. However, FORTA-C/D only detected a minority of low dose aspirin (20%), opioid (27.3%) and non-opioid analgesic (8.0%) PIMs. In comparison to FORTA-C/D, STOPP detected more low-dose-aspirin (86.7%) PIMs but fewer psycho-analeptic (4.7%) PIMs. Loop diuretic (69.4%) PIMs were exclusively detected by STOPP (69.4%) and STOPPFall (38.8%). EU(7)-PIM detected many more DOAC PIMs (98.1%) than any of the other tools (0% to 21.2%), and STOPP (80.0%) and STOPPFall (59.1%) detected more opioid PIMs than the other tools (0% to 27.3%).

PPOs. Table 6 shows that the 226 study participants had a total of 862 indications for medications listed in either FORTA-A or START. For 399 (46.3 (42.9–49.7)%) of these indications, a PPO was detected by one or both instruments. Of these, diabetes (12.8 (9.7–16.5)%) was the most commonly implicated indication, followed by hypertension (10.3 (7.5–13.7)%), chronic obstructive pulmonary disease (COPD) (8.8 (6.2–12.0)%), and atrial fibrillation (7.5 (5.1–10.6)%). The sensitivity of PPO detection (i.e., the proportion of all PPOs detected by each instrument) was similar, albeit somewhat higher, for START (60.9 (55.9–65.7)%) than for FORTA-A (51.9 (46.9–56.9). FORTA-A detected all hypertension - and diabetes PPOs, whereas START detected no hypertension PPOs (0.0%) and very few diabetes PPOs (3.9%). In contrast, START detected substantially more PPOs than FORTA-A for heart failure (100.0% vs 53.3%), depression (100.0% vs 0.0%), and atrial fibrillation (80.0% vs 30.3%).

#### 3.2.3. Overlaps between PIMs and PPO Instruments

**Figure 1:**
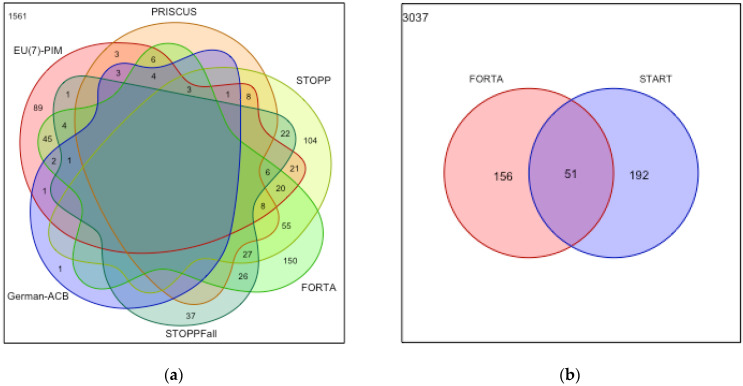
Venn diagrams of unique and overlapping detection of PIMs (**a**) and PPOs (**b**) by different tools. The numbers represent the quantities of PIMs/PPOs in that respective area (e.g., STOPP uniquely identified 104 PIMs, but 55 PIMs that STOPP detected were also detected by FORTA). Blank areas without numbers are to be interpreted as zero.

Figure 1a shows overlaps of the applied instruments in terms of detected PIMs. A total of 381 (58.8 (54.9–62.6)%) of all PIMs were exclusively detected by one instrument, 267 (41.2 (37.4–54.1)%) by two or more instruments, and 93 (14.4 (11.7–17.3)%) by three or more. A total of 150 (23.1%) PIMs were exclusively detected by FORTA C/D, 104 (16.0%) exclusively detected by STOPP, and 89 (13.7%) exclusively detected by EU(7)-PIM, while the German-ACB detected a single PIM exclusively and PRISCUS none. 

Figure 1b shows that of the 399 PPOs detected, a total of 51 (12.8%) were detected by both FORTA-A and START, whereas 156 (39.1%) were exclusively detected by FORTA-A and 192 (48.1%) exclusively by START. 

Except for moderate agreement between PRISCUS and German-ACB (Cohen’s Kappa 0.42 (0.23–0.59)), all other agreements were found to be poor, slight, or fair, as shown in Table 7 [28]. 

#### 3.2.4. Cumulative Sensitivity of Combining PIM Instruments

**Figure 2:**
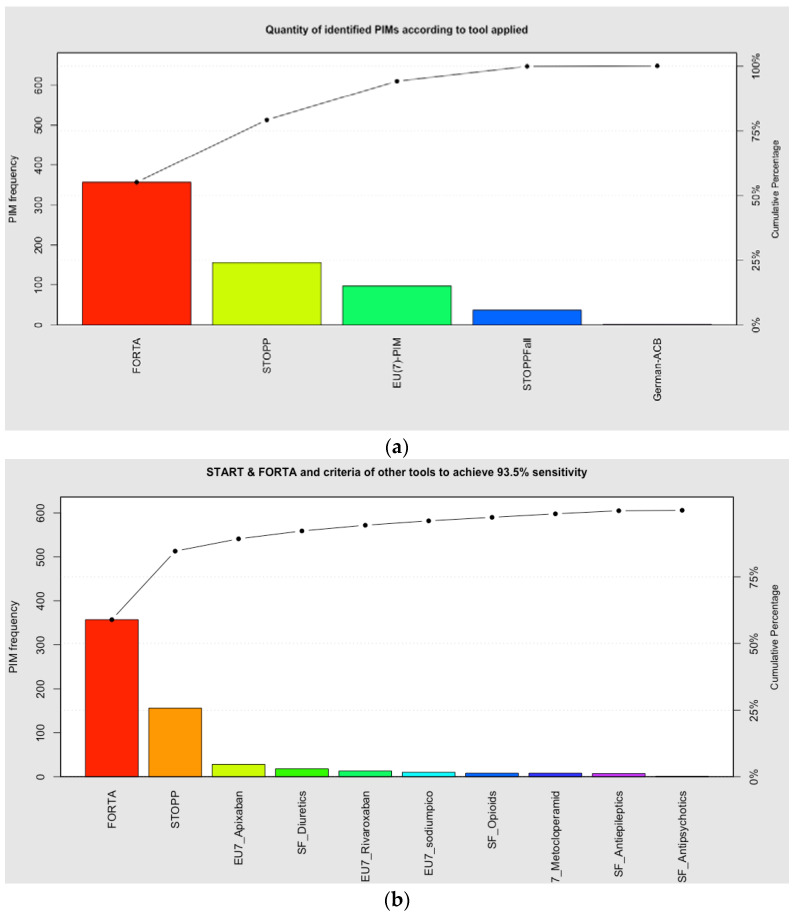
Pareto chart showing the number of distinct PIMs detected by PIM instruments in descending order of PIM prevalence, and the cumulative sensitivity of PIM detection achieved by adding distinct items from each instrument. Panel (**a**) shows the effects on the cumulative sensitivity of adding all distinct items from each instrument. Panel (**b**) shows the effects on the cumulative sensitivity of adding to FORTA C/D and STOPP the most prevalent PIMs from other tools.

The Pareto chart in Figure 2a shows (as bars) the percentage of all PIMs detected by FORTA-C/D, while the remaining bars show the percentage of new PIMs additionally detected by each tool, after application of the previous tool(s). The line shows the cumulative sensitivity (i.e., the percentage of PIMs detected) resulting from the addition of each tool. Since PRISCUS did not identify any PIMs exclusively, this tool was not considered in this analysis. Starting with FORTA C/D (which had the highest sensitivity, of 55.1%), adding STOPP achieves a cumulative sensitivity of 79.2%, and further adding EU(7)-PIM achieves a sensitivity of 94.1%. Figure 2b shows that after application of FORTA-C/D and STOPP, adding PIM criteria for four drugs (apixaban, rivaroxaban, and sodium picosulfate from the EU(7)-PIM list; diuretics from STOPPFall) increases the sensitivity by 10.6% to 89.8%. The addition of criteria relating to opioids, antiepileptics and antipsychotics (from STOPPFall), and metoclopramide (from EU(7)-PIM), increases the sensitivity further by 3.7% to 93.5%.

## 4. Discussion

### 4.1. Summary of Findings

This cross-sectional study of a convenience sample of 226 people in need of care, aged ≥ 65 years, in Bavaria (Germany) shows that the vast majority of participants received polypharmacy (92.5%). The vast majority (91.6%) also received at least one PIM after the application of six PIM tools together, with 79.6% receiving two or more PIMs, and over half (57.1%) receiving four or more PIMs. Similarly, most (82.7%) participants had at least one PPO considering FORTA-A and START together, and 50.0% had two or more PPOs. More than three quarters of the analyzed patients (76.1%) were affected by both PIMs and PPOs. 

No single PIM instrument reached full PIM coverage, and the detected PIM prevalence varied considerably by tool, ranging from 76.5% for FORTA C/D to 6.6% for German-ACB ≥ 3. Pairwise agreement between the PIM tools was poor to moderate and highest between PRISCUS and German-ACB (Cohen’s Kappa 0.42 (0.23–0.59)). FORTA C/D had the highest sensitivity of PIM detection (it identified 55.1% of all PIMs), and it also detected the most PIMs not identified by any other tool. However, stratification by drug group revealed that while FORTA-C/D had a high sensitivity for the detection of benzodiazepine, other psycholeptic, spironolactone, psychoanaleptic, and betablocker PIMs, it only detected a minority of low dose aspirin, opioid, and non-opioid analgesic PIMs. We found that combining items included in FORTA C/D and STOPP achieved a cumulative sensitivity of PIM detection of 79.2%, which could be further increased to 89.8% by additionally considering criteria relating to apixaban, rivaroxaban, and sodium picosulfate from the EU(7)-PIM list, and diuretics from STOPPFall. 

The PPO prevalence was similar for both instruments used (63.7% for START and 62.8% for FORTA A), but considerably lower than for their combined use (82.7%), consistent with each tool also identifying unique PIMs. While FORTA-A detected all hypertension and diabetes PPOs, START detected no hypertension PPOs (0.0%) and very few diabetes PPOs (3.9%), but substantially more PPOs than FORTA-A for heart failure (100.0% vs 53.3%), depression (100.0% vs 0.0%), and atrial fibrillation (80.0% vs 30.3%).

### 4.2. Comparison to Literature

Numerous previous studies have used several of the PIM and PPO tools used in this study to examine the PIM and/or PPO prevalence in different settings. According to a recent review of PIM prevalence studies [6], the proportions of study participants affected by PIMs was 44.3% for FORTA (vs. 76.5% in this study) and ranged from 26.7% to 67.3% for STOPP (vs. 65.9% in this study), from 37.5% to 90.6% for EU(7) PIM (vs. 61.9% in this study) and from 13.7% to 68.5% for PRISCUS (vs. 12.8% in this study). Campbell et al. (2010) found that 10.8% of a sample of African American adults aged ≥ 70 years were exposed to at least one drug with strong anticholinergic properties (vs. 6.6% in this study) [29,30]. The prevalence of PIMs according to STOPPFall was 85.4% in one study of hospitalized patients (vs. 36.3% in this study). According to the same review [6], the proportions of study participants affected by PPOs ranged from 19.8% to 64.2% for START (vs. 62.8% in this study). Compared to this data, this study of patients in need of care found the PIM prevalence to be at the high end for FORTA and STOPP/START, in the middle for EU(7)-PIM, and at the low end for PRISCUS, German-ACB and STOPPFall. This may reflect that PRISCUS is a German development, was published in 2010, and contributed to the EU(7) PIM list, while FORTA is a more recent development, and START/STOPP is less well known in the German setting. The discrepancy in the results for STOPPFall however is explained by differing measurement methods. While Damoiseaux-Volman et al. (2022) considered any use of STOPPFall medications as PIMs, we considered them as PIMs only if their users also had risk factors for falls specified in the STOPPFall deprescribing tool [31]. 

In contrast to prevalence studies using one tool, comparisons of two or more PIM or PPO tools in the same study population are much less common. In a Norwegian population of geriatric wards of people aged 65 or older taking one or more medication, the PIM prevalence was 62.4—69.2% for EU(7)PIM, which is comparable to our findings (61.9%) [32]. In a Kuwaiti population of primary care patients aged 65 years or older, the PIM prevalence was lower for FORTA (44.3%) than for STOPP (55.7%), which is in contrast to our findings (76.5% vs 65.9%, respectively) [33]. In a German population of 3189 Subjects, the PIM prevalence was highest for EU(7)PIM (70.1%), followed by FORTA (55.9%), and PRISCUS (24.7%), whereas in this study, FORTA-CD detected more PIMs (73.9%) than STOPP (65.9%) [34]. These findings highlight that the study population may not only influence the prevalence of polypharmacy, but also the relative performance of different instruments. 

### 4.3. Strengths and Limitations

To our knowledge, this is the first study to examine the sensitivity of PIM and PPO detection considering PIM and PPO instruments alone and in combination, which we considered most relevant to the German setting. Our analysis sheds light on the prevalence of PIMs and PPOs in a vulnerable population in need of care, which is often under-represented in clinical research. We were able to collect a comprehensive data set, which enabled us to apply the vast majority of items included in each tool. However, a small number of items (19 items from the STOPP tool) could not be applied due to missing data, implying that the detected prevalence may be an underestimation. The main limitations of this study are its relatively small sample size and the potential selection bias resulting from convenience sampling. Nevertheless, study participants were included from a variety of settings, and our sample included study participants irrespective of their physical or mental health, or their cognitive abilities.

### 4.4. Implications for Clinical Practice and Research

Our findings demonstrate a very high prevalence of PIMs and PPOs among this vulnerable sample of patients in need of care, with the vast majority of study participants affected by PIM, PPO, or both. These findings alone reinforce the need to regularly and comprehensively review all medications these patients are taking. Our findings suggest that using a single tool may leave a substantial number of PIMs and PPOs undetected, but that by combining FORTA-C/D and STOPP, as well as FORTA-A and START, into comprehensive tools, the proportion of detectable PIMs and PPOs can be considerably increased. Nevertheless, it is clear that any combination of PIM tools applied without computerized support may not comprehensively detect all medication risks associated with polypharmacy, given the vast number of possible drug-drug and drug-disease interactions. 

It is also clear that detection of PIMs and PPOs alone does not suffice to improve patient outcomes, which additionally requires clinical judgment to identify actually inappropriate medication, as well as effective interventions to overcome barriers to PIM deprescribing. This study has examined how the sensitivity of PIM and PPO detection can be enhanced in older people in need of care by combining prominent PIM and PPO instruments, but our findings should be confirmed in other settings. In addition, our findings should be supplemented by research characterizing the extent to which PIM and PPO tools identify medication that actually requires medication changes (i.e., deprescribing or initiation of drugs), which interventions may overcome pertinent barriers to which medication changes, as well as the effects of such changes on outcomes that matter to patients. 

## 5. Conclusions

Instruments which explicitly highlight common and clinically relevant potentially inadequate medication (PIM) and/or potential prescribing omissions (PPOs) may support clinicians in identifying targets for medicines optimization among older people with polypharmacy. However, this study shows that PIM and PPO instruments differ considerably, both in terms of the quantity and nature of medication related problems they detect, and that it therefore matters which tool is used in which setting. Our study also demonstrates that using a single existing tool may not have sufficient sensitivity to detect PIMs and PPOs, and that combining distinct items from two or more instruments may considerably increase the sensitivity. Further research is required to optimize the composition of PIM and PPO screening instruments in terms of both the sensitivity and specificity in different settings. 

## Figures and Tables

**Table 1:** Description of applied PIM and PPO tools.

Instruments	Last Update	Geographical Origin	Description of Structure	Development Methods	No. of Items	Data Types
PIM tools						
Patient-in-focus listing approach (PILA)						
FORTA	2021	Germany	List of the most frequently used pharmaceuticals in Germany, presented in respect to indication groups. Classification in four classes (A-D), where C—questionable and D—avoid contain potentially inadequate medication.	DELPHI	299 substances used in 30 indication groups	diagnosis, medication
STOPP	2014	UK/ Ireland	Screening tool by organ and functional system to identify potentially inadequate medication.	DELPHI	80 criteria	diagnosis, vital signs, lab data, patient history, medication
STOPPFall	2020	EU/ Finland	After 14 medication classes were defined as case-risk-increasing-drug (FRID), condition-based criteria for PIM classification were created in a deprescribing tool.	DELPHI	14 medication classes with 56 criteria in total	diagnosis, medication
Drug-oriented list approach (DOLA)						
PRISCUS	2011	Germany	Negative list, which includes PIM as well as recommendation for substitution of these.	DELPHI	83 substances	medication
EU(7)-PIM	2015	Europe	Developed by a Commission including experts from seven European countries; includes the PRISCUS list in its entirety.	DELPHI	282 substances	medication
German-ACB	2018	Germany	Substances classified as anticholinergic active are scored into three activity levels (1–3). The anticholinergic burden of each patient is defined as the sum of their individual anticholinergic activity levels.	systematic literature research	507 substances, thereof 151 with anticholinergic activity	medication
PPO tools						
FORTA	2021	Germany	Classification in four classes (A-D), where A—indispensable and B—beneficial contain potentially necessary medication.	DELPHI	299 substances used in 30 indication groups	diagnosis, medication
START	2014	UK/ Ireland	Screening tool by organ and functional system to identify potentially necessary medication.	DELPHI	34 criteria	diagnosis, vital signs, lab data

**Table 2:** Baseline characteristics of the study population (*n* = 226).

Characteristics	*n* (%) [95%-CI]
Age (Years)	
median (Q1–Q3)	84 (80–89)
65–79	53 (23.5) [18.0–92.6]
80–89	122 (54.0) [47.2–60.6]
≥90	51 (22.6) [17.3–28.6]
Sex	
Female	161 (71.2) [64.9–77.0]
Male	65 (28.8) [23.0–35.1]
nursing situation (*n* = 213)	
long-term care facility (LTCF)	159 (74.6) [68.3–80.3}
outpatient care	35 (16.4) [11.7–22.1]
number of medications (*n* = 226)	
median (Q1–Q3)	9.00 (7.0–12.0)
≥5 (polypharmacy)	209 (92.5) [88.2–95.6]
≥10 (excessive polypharmacy)	103 (45.6) [39.0–52.3]
Charlson Comorbidity Index (CCI) (*n* = 226)	
median (Q1–Q3)	3 (1.0–5.0)
moderate comorbidity (3–4 pts.)	54 (23.9) [18.5–30.0]
severe comorbidity (≥5 pts.)	59 (26.1) [20.5–32.0]
Six-Item-Screener (*n* = 201)	
median number of errors (Q1–Q3)	1 (0–2)
cognitive impaired (≥3 errors)	49 (24.3) [18.6–30.9]
MoCA—BLIND ^1^ (*n* = 169)	
median (Q1–Q3)	17.0 (14.0–20.0)
mild cognitive impairment (≤17)	79 (53.3) [45.4–61.0]
7-Point Clinical Frailty-Scale (*n* = 108)	
median (Q1–Q3)	6.0 (5.0–7.0)
mild to moderately frail (5–6 pts.)	49 (45.4) [35.8–55.2]
severely to very severely frail (7–8 pts.)	41 (38.0) [28.8–47.8]
distribution of chronic diseases (*n* = 226)	
hypertension (*n*)	170 (75.2) [69.1–80.7]
dyslipidemia (*n*)	63 (27.9) [22.1–34.2]
diabetes (*n*)	61 (27.0) [21.3–33.3]
heart failure (*n*)	53 (23.5) [18.1–29.5]
hypothyroidism (*n*)	44 (19.5) [14.5–25.2]
depression (*n*)	48 (21.2) [16.1–27.2]
atrial fibrillation (*n*)	72 (31.9) [25.8–38.4]
chronic obstructive pulmonary disease (COPD) (*n*)	39 (17.3) [12.6–22.8]
Parkinson’s disease (*n*)	17 (7.5) [4.4–11.8]
coronary heart disease	39 (17.3) [12.6–22.8]
stroke	16 (7.1) [4.1–11.2]
renal failure	45 (19.9) [14.9–25.7]

^1^ Montreal Cognitive Assessment BLIND; Q1—percentile 25, Q3—percentile 75; 95%-CI—95% confidence interval calculated with the exact binomial test.

**Table 3:** Prevalence of potentially inappropriate medication (PIM) within the study population (*n* = 226).

No. of Detected PIMs per Patient	Tools Applied						
	All	FORTA-C/D	STOPP	EU(7)-PIM	PRISCUS	STOPPFall	German-ACB
	PIM Count (*%*) [95%-CI]						
≥1	207 (91.6)	173 (76.5)	149 (65.9%)	140 (61.9%)	29 (12.8%)	82 (36.3%)	15 (6.6%)
	[87.2–94.9]	[70.5–81.9]	[59.4–72.1]	[55.3–68.3]	[8.8–17.9]	[30.0–42.9]	[3.8–10.7]
1	27 (11.9)	70 (31.0%)	73 (32.3%)	83 (36.7%)	22 (9.7%)	50 (22.1%)	14 (6.2%)
	[8.0–16.9]	[25.0–37.4]	[26.3–38.9]	[30.4–43.4]	[6.2–14.4]	[16.9–28.1]	[3.4–10.2]
2	27 (11.9)	47 (20.8%)	44 (19.5%)	34 (15.0%)	7 (3.1%)	24 (10.6%)	1 (0.4%)
	[8.0–16.9]	[15.7–26.7]	[14.5–25.2]	[10.6–20.4]	[1.3–6.3]	[6.9–15.4]	[0.0–2.4]
3	24 (10.6)	37 (16.4%)	19 (8.4%)	17 (7.5%)	0 (0.0)	6 (2.7%)	0 (0.0)
	[6.9–15.4]	[11.8–21.9]	[5.1–12.8]	[4.4–11.8]	[0.0–1.6]	[1.0–5.7]	[0.0–1.6]
≥4	129 (57.1)	19 (8.4%)	13 (5.8%)	6 (2.7%)	0 (0.0)	2 (0.9%)	0 (0.0)
	[50.3–63.6]	[5.1–12.8]	[3.1–9.6]	[1.0–5.7]	[0.0–1.6]	[0.1–3.2]	[0.0–1.6]

**Table 4:** Prevalence of potentially inappropriate medication (PIM) within the study population (*n* = 226).

No. of Detected PPOs per Patient	Tools Applied		
	All	FORTA-A	START
	PPO Count (*%*) [95%-CI]		
≥1	187 (82.7)	142 (62.8)	144 (63.7)
	[77.2–87.4]	[56.2–69.1]	[57.1–69.9]
1	74 (32.7)	94 (41.6)	79 (35.0)
	[26.7–39.3]	[35.1–48.3]	[28.8–41.6]
2	56 (24.8)	33 (14.6)	32 (14.2)
	[19.3–30.9]	[10.3–19.9]	[9.9–19.4]
3	34 (15.0)	13 (5.8)	22 (9.7)
	[10.6–20.4]	[3.1–9.6]	[6.2–14.4]
≥4	23 (10.2)	2 (0.1)	11 (4.9)
	[6.6–14.9]	[0.1–3.2]	[2.5–8.5]

**Table 5:** Sensitivity of PIM detection for each tool and stratified by the most commonly implicated drugs (accounting for 67.6% of all PIMs detected by any tool).

Medication	Tools Applied						
	All	FORTA-C/D	STOPP	EU(7)-PIM	PRISCUS	STOPPFall	German-ACB
	PIM Count (% of All PIMs) [95%-CI]						
all	648 (100.0)	357 (55.1)	275 (42.4)	226 (34.9)	36 (5.6)	124 (19.1)	16 (2.5)
	[100.0–100.0]	[51.2–59.0]	[38.6–46.3]	[27.3–34.0]	[3.9–7.6]	[16.2–22.4]	[1.4–4.0]
acetylsalicylic acid	15 (2.3)	3 (20)	13 (86.7)	1 (6.7)	0 (0.0)	0 (0.0)	0 (0.0)
	[1.3–3.8]	[4.3–48.1]	[59.5–98.3]	[0.2–32.0]	[0.0–21.8]	[0.0–21.8]	[0.0–21.8]
direct oral anticoagulants	52 (8.0)	0 (0.0)	11 (21.2)	51 (98.1)	0 (0.0)	0 (0.0)	0 (0.0)
	[6.1–10.4]	[0.0–6.8]	[11.1–34.7]	[89.7–99.9]	[0.0–6.8]	[0.0–6.8]	[0.0–6.8]
beta blocker	47 (7.3)	35 (74.5)	17 (36.2)	3 (6.4)	0 (0.0)	0 (0.0)	0 (0.0)
	[5.4–9.5]	[59.7–86.1]	[22.7–51.5]	[1.3–17.5]	[0.0–7.5]	[0.0–7.5]	[0.0–7.5]
benzodiazepines	14 (2.2)	14 (100.0%)	14 (100.0%)	5 (35.7%)	3 (21.4%)	2 (14.3%)	0 (0.0%)
	[1.2–3.6]	[76.8–100.0]	[76.8–100.0]	[12.8–64.9]	[4.7–50.8]	[1.8–42.8]	[0.0–2.3]
other psycholeptics	82 (12.7)	66 (80.5)	80 (97.6)	21 (25.6)	6 (7.3)	32 (39.0)	1 (1.2)
	[10.2–15.5]	[70.3–88.4]	[91.5–99.7]	[15.6–35.1]	[2.7–15.2]	[28.4–50.4]	[0.0–6.6]
psychoanaleptics	85 (13.1)	82 (96.5)	4 (4.7)	24 (28.2)	8 (9.4)	15 (17.7)	7 (8.2)
	[10.6–16.0]	[90.0–99.3]	[1.3–11.6]	[19.0–39.0]	[4.2–17.8]	[10.2–27.4]	[3.4–16.2]
opioids	44 (6.8)	12 (27.3)	31 (70.5)	4 (9.1)	0 (0.0)	26 (59.1)	0 (0.0)
	[5.0–9.0]	[15.0–42.8]	[54.8–0.83]	[2.5–21.7]	[0.0–8.0]	[43.2–73.7]	[0.0–8.0]
non-opiod analgetics	25 (3.9)	2 (8.0)	24 (96.0)	9 (36.0)	3 (12.0)	0 (0.0)	0 (0.0)
	[2.5–5.6]	[1.0–26.0]	[79.6–99.9]	[18.0–57.5]	[2.5–31.2]	[0.0–13.7]	[0.0–13.7]
loop diuretics	49 (7.6)	0 (0.0)	34 (69.4)	0 (0.0)	0 (0.0)	19 (38.8)	0 (0.0)
	[5.6–9.9]	[0.0–7.3]	[54.6–81.7]	[0.0–6.5]	[0.0–7.3]	[25.2–53.8]	[0.0–7.3]
spironolactone	25 (3.9)	25 (100.0)	0 (0.0)	6 (24.0)	6 (24)	4 (16.0)	0 (0.0)
	[2.5–5.6]	[86.3–100.0]	[0.0–13.7]	[9.4–45.1]	[9.4–45.1]	[4.5–36.1]	[0.0–13.7]

**Table 6:** Sensitivity of PPO detection for each tool, stratified by the most commonly implicated indications (accounting for 49.9% of all PPOs detected by any tool).

Indications	Tools Applied		
	All	FORTA-A	START
	PPO Count (% of All PIMs) [95%-CI]		
all	399 (100.0)	207 (51.9)	243 (60.9)
	[100.0–100.0]	[46.9–56.9]	[55.9–65.7]
Hypertension	41 (10.3)	41 (100.0)	0 (0.0)
	[7.5–13.7]	[91.4–100.0]	[0.00–8.6]
Diabetes	51 (12.8)	51 (100.0)	2 (3.9)
	[9.7–16.5]	[93.0–100.0]	[0.5–13.5]
Dyslipidemia	2 (0.5)	0 (0.0)	2 (100.0)
	[0.1–1.8]	[0.0–84.2]	[15.8–100.0]
Heart failure	15 (3.8)	8 (53.3)	15 (100.0)
	[2.1–6.1]	[26.6–78.7]	[78.2–100.0]
Hypothyroidism	1 (0.3)	1 (100.0)	0 (0.0)
	[0.0–1.4]	[2.5–100.0]	[0.0–97.5]
Depression	21 (5.3)	0 (0.0)	21 (100.0)
	[3.3–7.9]	[0.0–16.1]	[83.9–100.0]
Atrial fibrillation	30 (7.5)	10 (30.3)	24 (80.0)
	[5.1–10.6]	[17.3–52.8]	[61.4–92.3]
Chronic obstructive pulmonary disease	35 (8.8)	26 (74.3)	26 (74.3)
	[6.2–12.0]	[56.7–87.5]	[56.7–87.5]
Parkinson’s disease	3 (0.8)	3 (100.0)	3 (100.0)
	[0.2–2.2]	[29.2–100.0]	[29.2–100.0]

**Table 7:** Pairwise agreement (Cohen’s Kappa) of PIM detection via five different tools.

	FORTA	STOPP	EU(7)-PIM	PRISCUS	STOPPFall
FORTA	-	-	-	-	-
STOPP	0.27 (0.22–0.33)	-	-	-	-
EU(7)-PIM	0.25 (0.19–0.30)	0.17 (0.12–0.23)	-	-	-
PRISCUS	0.08 (0.04–0.12)	0.10 (0.06–0.15)	0.25 (0.19–0.32)	-	-
STOPPFall	0.20 (0.15–0.25)	0.21 (0.16–0.27)	0.00 (−0.04–0.03)	−0.03 (−0.03–0.02)	-
German-ACB	0.04 (0.01–0.07)	0.02 (−0.01–0.04)	0.11 (0.06–0.16)	0.42 (0.23–0.59)	0.00 (−0.03–0.03)

Interrater reliability estimated by Cohen’s Kappa. Agreement classification used: poor (<0.00), slight (0.00–0.20), fair (0.21–0.40), moderate (0.41–0.60), substantial (0.61–0.80), almost perfect (0.81–1.00).

## Data Availability

The data presented in this study are available on request from the corresponding author. The data are not publicly available due to data protection reasons.
